# mtRF1a Is a Human Mitochondrial Translation Release Factor Decoding the Major Termination Codons UAA and UAG

**DOI:** 10.1016/j.molcel.2007.06.031

**Published:** 2007-09-07

**Authors:** Hamid Reza Soleimanpour-Lichaei, Inge Kühl, Mauricette Gaisne, Joao F. Passos, Mateusz Wydro, Joanna Rorbach, Richard Temperley, Nathalie Bonnefoy, Warren Tate, Robert Lightowlers, Zofia Chrzanowska-Lightowlers

**Affiliations:** 1 Mitochondrial Research Group, Newcastle University, Framlington Place, Newcastle upon Tyne NE2 4HH, UK; 2 Centre de Génétique Moléculaire, CNRS Batiment 26, Avenue de la Terrasse, 91198 Gif sur Yvette Cedex, France; 3 Department of Biochemistry, University of Otago, P.O. Box 56, 710 Cumberland Street, Dunedin 9016, New Zealand; 4 Centre for Integrated Systems Biology of Ageing and Nutrition, Newcastle University, Newcastle upon Tyne NE4 6BE, UK

**Keywords:** RNA

## Abstract

Human mitochondria contain their own genome, encoding 13 polypeptides that are synthesized within the organelle. The molecular processes that govern and facilitate this mitochondrial translation remain unclear. Many key factors have yet to be characterized—for example, those required for translation termination. All other systems have two classes of release factors that either promote codon-specific hydrolysis of peptidyl-tRNA (class I) or lack specificity but stimulate the dissociation of class I factors from the ribosome (class II). One human mitochondrial protein has been previously identified in silico as a putative member of the class I release factors. Although we could not confirm the function of this factor, we report the identification of a different mitochondrial protein, mtRF1a, that is capable in vitro and in vivo of terminating translation at UAA/UAG codons. Further, mtRF1a depletion in HeLa cells led to compromised growth in galactose and increased production of reactive oxygen species.

## Introduction

A central mitochondrial function is that of oxidative phosphorylation, the process of coupling respiration to ATP production. Thirteen of the polypeptides critical for this function are encoded by the mitochondrial genome (mtDNA). Mitochondrial protein synthesis is therefore central to the life of aerobic eukaryotic cells; however, our understanding of mammalian mitochondrial gene expression is far from complete, particularly concerning the mechanisms governing mitochondrial protein synthesis. It is essential that we redress this situation, as it has become increasingly clear that defects of mitochondrial translation constitute an important subset of mitochondrial disorders (Coenen et al., 2004; Jacobs and Turnbull, 2005; Miller et al., 2004; Smeitink et al., 2006; Valente et al., 2007).

The mitochondrial translation apparatus employs a different codon usage from the universal code, with some species decoding AUA as methionine and the majority converting the universal STOP codon UGA into a tryptophan. Thus, most mitochondrial translation systems have reduced the number of termination codons from three to two (Elzanowski and Ostell, 2000). Although the human mitochondrial code also employs these two changes, it varies still further by commandeering two additional termination codons, AGA and AGG, that would conventionally encode arginine (Barrell et al., 1979). Once the translation complex has reached a termination codon, the completed protein must be dissociated from the final tRNA, the ribosome, and its cognate mRNA. The proteins responsible for fulfilling these functions are the release factors (RFs), of which there are two classes (for review, see Kisselev et al., 2003; Poole and Tate, 2000). Class I is codon specific with peptide motifs that distinguish the STOP codons. In prokaryotic RFs, this is an SPF or PXT tripeptide motif (Ito et al., 2000; Nakamura et al., 2000). There is also a GGQ loop/motif facilitating the termination of translation by hydrolysis of the ester bond between the tRNA and the nascent polypeptide (Frolova et al., 1999; Seit-Nebi et al., 2001). Eubacterial and eukaryotic cytosolic release factors differ in their class I constituents. Eubacteria utilize three STOP codons and require two release factors, RF1 and RF2. Both recognize UAA, but RF1 also interacts with UAG and RF2 with UGA. Thus, although each bacterial RF recognizes a pair of STOP codons, both are necessary for appropriate translation termination of the entire mRNA population. Most eukaryota and archaebacteria utilize the same three stop codons (UAA, UAG, and UGA), but each organism requires only a single omnipotent protein, named eRF1 or aRF1, respectively, that can recognize all three STOP triplets (Frolova et al., 1999; Konecki et al., 1977). This leaves the intriguing question of whether human mitochondria resemble more closely the eukaryota and archaebacteria and have evolved a single release factor that can recognize all four codons that are used, or whether they follow the eubacterial paradigm of two RFs, each recognizing a pair of stop codons, which would more closely reflect their α-proteobacterial origins.

Thus far, no human mitochondrial release factor has been characterized. A candidate, mtRF1, was proposed several years ago from bioinformatic analyses (Zhang and Spremulli, 1998) and has since been assimilated into the scientific literature (for example, Antonicka et al., 2006; Askarian-Amiri et al., 2000; Bulygin et al., 2002; Chavatte et al., 2003; Frolova et al., 2002; Kisselev et al., 2003; Oparina et al., 2005; Petry et al., 2005; Smeitink et al., 2006). Class I release factors of the RF1 type contain a PXT tripeptide motif responsible for codon recognition. Human mtRF1, however, exhibits a hexapeptide PEVGLS across this region.

Here we report that we have now identified an alternative gene product with greater similarity across the tripeptide recognition motif of the bacterial RF1. Moreover, we ascribe mitochondrial localization and release activity to this protein, mtRF1a, which exhibits specific recognition of the main codons used, UAA and UAG, consistent with it being a release factor in human mitochondria.

## Results

### Human mtRF1 Is a Mitochondrial Protein

**Figure 1:**
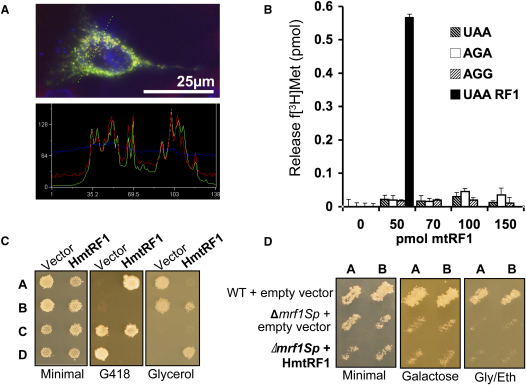
Human mtRF1 Is a Mitochondrial Protein with No Detectable Release Factor Activity (A) Human mtRF1 is targeted to mitochondria. HeLa cells were transiently transfected for 24 hr with a construct expressing 290 N-terminal residues of mtRF1 fused to GFP. Cells were also stained to visualize mitochondria (mitotracker CMH_2_X-Ros) and nuclei (DAPI). Fluorescence images were captured and mitochondrial colocalization of the fusion protein confirmed by superimposition of the green and red signals in a linescan of the image (line visible in upper panel). An image typical of the three independent transfections is shown. (B) Purified mtRF1 does not induce translation termination in vitro. Human mtRF1 lacking the N-terminal 49 residues was purified and used to assess translation termination from 5 pmol ribosomes programmed with the synthetic codon (400 pmol) indicated. Release factor activity was measured by the hydrolysis of f[^3^H]met from its cognate tRNA^Met^ as detailed in the Supplemental Experimental Procedures. Nonlimiting amounts of both *E. coli* RF1 (50 pmol) and UAA triplet (400 pmol) were used in tandem as a positive control for the assay. Standard errors were calculated from a minimum of eight repeats. (C) Human mtRF1 fails to restore respiratory competence in *S. cerevisiae Δmrf1*. Diploids producing human mtRF1 or containing vector alone were generated from the *Δmrf1* strain crossed to the wild-type CW252/A. Following sporulation, asci were dissected and resultant haploid sister spores (A–D) assessed for presence of the plasmid (−uracil), resistance to geneticin G418 showing deletion of *MRF1Sc*, and growth on a nonfermentable carbon source (glycerol). (D) Human mtRF1 fails to restore respiratory competence in *S. pombe Δmrf1*. The new fission yeast strain devoid of endogenous mitochondrial release factor NB329 (Experimental Procedures) and its isogenic wild-type strain NB205-6A were transformed with either empty vector or one producing human mtRF1. Transformants were patched on uracil-free minimal medium selecting for the plasmid, replicated onto complete galactose or glycerol plates, and incubated at 28°C.

A number of papers now state that human mitochondria have a single release factor, mtRF1, which was identified *in silico* a number of years ago (Zhang and Spremulli, 1998). To identify whether this protein is indeed mitochondrial, we generated a GFP fusion construct C-terminal to the predicted mtRF1 N-terminal targeting sequence. HeLa cells were transiently transfected and after 24 hr were treated with mitotracker-CMH_2_X ROS to visualize the mitochondria. Concordance of the fluorescence signals indicated mitochondrial localization of the fusion protein (Figure 1A).

### Does Human mtRF1 Function as a Release Factor?

To assess whether mtRF1 could function as a release factor, we adopted an in vitro and an in vivo approach. In vitro release factor assays were performed essentially as established by Caskey and colleagues (Caskey et al., 1971). Briefly, ribosomes were incubated with AUG synthetic triplet and f[^3^H]Met-tRNA^Met^ to load the ribosomal P site prior to addition of purified mtRF1 and the synthetic stop codon to be assessed. Free f[^3^H]Met was then quantified to indicate release activity. The exact size of the N-terminal presequence that might be cleaved from the full-length protein upon mitochondrial import is unknown, and predictions from targeting programmes vary from 17 up to 70 residues. Moreover, sequence alignment indicates that the first 63 amino acids of the mtRF1 N-terminal extension do not align with the bacterial RF1. Therefore, to simulate possible mature polypeptides without the loss of recognized functional domains, two proteins carrying N-terminal truncations and increased solubility, mtRF1Δ49 and mtRF1Δ69 (data not shown), were purified. These were tested against the universal and mitochondrial-specific stop codons, but no activity could be detected with UAA, AGA, or AGG codons (Figure 1B).

For in vivo studies, we assessed whether human mtRF1 could suppress the phenotype caused by loss of the endogenous release factor from yeast. Mutations of *S. cerevisiae MRF1* cause a respiratory dysfunction associated with the loss of intact mtDNA (Pel et al., 1992). Such mtDNA instability is known to result from defects in the general mitochondrial translation machinery (Myers et al., 1985). Expression of the entire human *mtRF1* cDNA in a heterozygous Δ*mrf1 rho^+^* diploid, followed by sporulation, did not yield any *Δmrf1* spores that could grow on nonfermentable carbon sources (glycerol, Figure 1C). Similarly, there was no restoration of cytochrome spectra, consistent with a lack of mitochondrial protein synthesis and respiratory complex assembly (data not shown). In fact, the *Δmrf1* spores producing human mtRF1 could still not maintain their mtDNA. This raised the possibility that complementation occurred but was too weak to allow mtDNA maintenance, thus preventing nonfermentable growth. To address this problem, a new *mrf1*-deleted strain was engineered in the fission yeast *Schizosaccharomyces pombe*, a *petite*-negative yeast, for which mtDNA loss events are highly counterselected. The Δ*mrf1Sp* strain showed reduced growth at 28°C on galactose, which forces respiration, and it stopped growing at 36°C on the same medium (Figure 1D, see Figure S4B in the Supplemental Data available with this article online) but retained mtDNA (Figure S1). Transfectants of this strain expressing the cDNA encoding human mtRF1 were also unable to respire, and there was no restoration of growth on glycerol/ethanol or galactose, as shown in Figure 1D, despite the presence of an intact mtDNA.

### Identification of an Alternative Candidate Human Mitochondrial Release Factor

The lack of release activity in vitro could have been due to the necessity to use the heterologous bacterial 70S ribosomes rather than 55S mitoribosomes. To date, it has not been possible to establish a reproducible in vitro assay with mammalian mitoribosomes, unlike with yeast components (Askarian-Amiri et al., 2000), perhaps explained by recent cryo-electron microscopy studies, which have revealed that isolated mammalian mitoribosomes retain deacylated mt-tRNA in the P site (Sharma et al., 2003). Second, the failure of mtRF1 to restore respiration in either of the yeast Δ*mrf1* strains may be explained by yeast Mrf1p recognizing only UAA/UAG as termination codons (as all yeast mt-mRNAs contain UAA or UAG as stop codons), while those recognized by the human mtRF1 could potentially be AGG and AGA. If this is the case, then there may be a second human mitochondrial release factor. Searching of the human databases revealed a second candidate that we will call mtRF1a (UniprotKB/TrEMBL Q96EX4). This predicted polypeptide of 380 amino acids shows an overall sequence identity of approximately 42% with mtRF1, 34% with yeast mRFs (*S. pombe*, *S. cerevisiae*, *K. lactis*), 40% with bacterial RF1s, and 41% similarity to the RF from a close evolutionary relative *R. prowazeckii* (Andersson et al., 1998; Gray, 1998) (Figure S2). Moreover, mtRF1a contains a PXT motif that more closely resembles other RF1s, containing the triamino acid recognition motif PKT, rather than the hexapeptide sequence in mtRF1 (Table 1).

**Figure 2:**
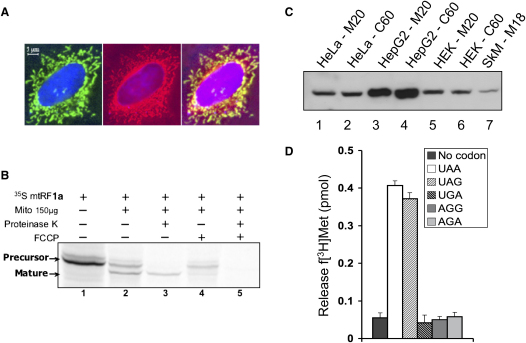
Human mtRF1a Is a Mitochondrial Release Factor that Recognizes UAA and UAG Codons (A) Human mtRF1a is targeted to mitochondria. HeLa cells were transiently transfected (24 hr) with a construct expressing 357 N-terminal residues of mtRF1a fused to GFP. Cells were also stained to visualize mitochondria (mitotracker CMH_2_X-Ros) and nuclei (DAPI). Fluorescence images were captured and mitochondrial colocalization of the fusion protein confirmed by superimposition of the green (fusion protein) and red (mitochondrial) signals as shown in the far right panel. The image shown is representative of three independent repeats. (B) Human mtRF1a is imported into mitochondria and matured. Full-length ^35^S-radiolabeled mtRF1a was in vitro synthesized (lane 1) and incubated with rat liver mitochondria. Under import conditions, two products are visible, the full-length preprotein and the mature protein (lane 2). Components (FCCP, Proteinase K) were added as indicated. (C) Human mtRF1a is found in the mitochondria of all cell lines and tissues analyzed. Human Hep G2, HEK293, and HeLa cell (C60 μg) or mitochondrial (M20 μg) lysates along with human skeletal muscle mitochondria (SkM–M18 μg) were prepared and subjected to western blot analysis with anti-mtRF1a antibodies. A single protein of approximately 40 kDa was visible in all cell/tissue types tested and found exclusively in the mitochondrial fraction (not in postmitochondrial supernatants, data not shown). No crossreactivity was noted with purified mtRF1 (data not shown). (D) Human mtRF1a has release factor activity with UAA and UAG, but not UGA codons. Release assays were performed with 50 pmol mtRF1a and 5 pmol ribosomes, as detailed in the Experimental Procedures. Ribosomes were programmed independently with five triplet codons (400 pmol), four of which are termination codons in human mitochondria (UAA/G, AGA/G), and UGA that encodes tryptophan in mitochondria but acts as a termination codon in the cytosol. No codon controls were also performed. Standard errors were calculated from a minimum of three or a maximum of 11 repeats.

As with mtRF1, a C-terminal GFP fusion protein construct was generated for mtRF1a and transiently transfected into HeLa cells before staining with mitotracker after 24 hr. Mitochondrial targeting was shown by colocalization of the fluorescence signals (Figure 2A). To confirm intramitochondrial uptake, import experiments were performed with in vitro-translated ^35^S-labeled mtRF1a and isolated mitochondria (Figure 2B and Supplemental Experimental Procedures). Import was shown to be dependent on a mitochondrial membrane potential (FCCP, lanes 4 and 5) and resulted in cleavage of the presequence (lanes 2 and 3). The matured protein was resistant to added Proteinase K, confirming mitochondrial uptake (lane 3).

Any factor involved in mitochondrial protein translation would be predicted to be present in all cell types. Antisera were raised against purified recombinant mtRF1a and affinity purified. Mitochondrial protein isolated from human skeletal muscle and a variety of human cell lines were analyzed by western analysis, and mtRF1a was found to be ubiquitous (Figure 2C). Further, by comparison of HeLa extracts against a standard curve of purified mtRF1a, we were able to calculate approximately 30,000 molecules per cell (data not shown).

### Human mtRF1a Is a Translation Release Factor

Purified recombinant mtRF1a was tested in the termination assay described above. Initial assays showed release of f[^3^H]Met when either UAA or UAG was used as a synthetic stop codon, confirming that mtRF1a could function as a release factor. Assay conditions were optimized for the human mitochondrial release factor (Figure S3). Using these assay parameters, the human recombinant mtRF1a was interrogated with a full panel of codons. Figure 2D clearly demonstrates that mtRF1a mediated codon-specific release of f[^3^H]Met from the termination complex. There is significant recognition of UAA and UAG stop codons by human mtRF1a but only residual noncatalytic levels comparable to those obtained when no codon was present for the noncognate stops AGA and AGG. Crucially, the UGA codon, encoding tryptophan in the human mitochondrion, did not stimulate release activity. Elongating the stop codon 3′ to include three further adenosine residues to mimic the context with the poly(A) tail made no significant change to the release activity observed.

Although the human mtDNA utilizes four triplet sequences as stop signals, the usage is not evenly distributed at the end of the 13 mitochondrial open reading frames (ORFs) (Anderson et al., 1981). The noncanonical AGA and AGG stops are used only once each, for *MTCOI* and by *MTND6*, respectively. Of the remaining 11 ORFs, *MTCO2* and *MTATP8* employ UAG as stop codons, and the final nine all use UAA encoded directly by the genome or completed by the addition of a poly(A) tail. Thus, 11 of the 13 transcripts utilize stop signals recognized by human mtRF1a.

**Figure 3:**
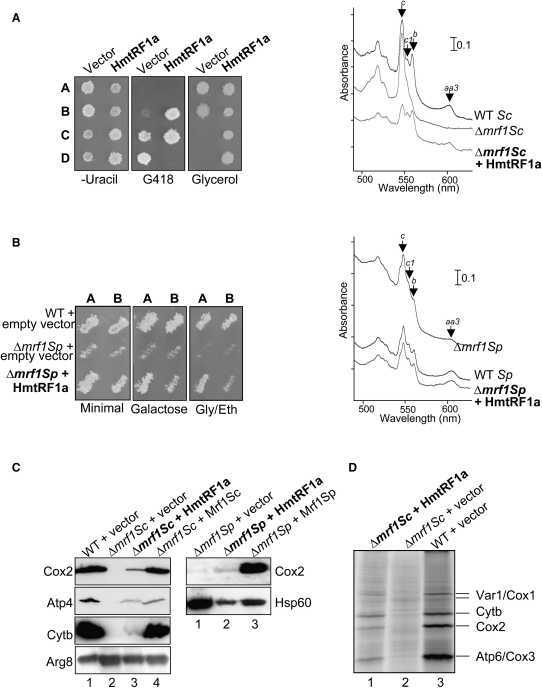
Human mtRF1a Can Suppress the Respiratory Deficiency in *Δmrf1* Budding and Fission Yeast (A) Human mtRF1a restores respiratory growth of *S. cerevisiae* lacking a mitochondrial release factor. The heterozygous Δ*mrf1* diploid strain (Figure 1C) was transfected with empty vector or encoding human mtRF1a prior to sporulation and tetrad analysis as described. The right panel shows low-temperature spectra of cells grown on glucose (Δ*mrf1*) or glycerol (WT and Δ*mrf1* + HmtRF1a) medium. (B) Human mtRF1a can function in fission yeast mitochondria. The *Δmrf1Sp* strain NB329 or the isogenic wild-type was transformed with either control vector or one producing mtRF1a. Transformants patched on uracil-free medium were replicated onto galactose or glycerol/ethanol medium and incubated at 28°C. Spectral analysis from Δ*mrf1* cells (glucose), WT, and Δmrf1cells expressing *MTRF1a* (galactose) is shown in the right panel. (C) Human mtRF1a partially restores the level of mitochondrial gene products in yeast. (Left panel) Western analysis of *S. cerevisiae* respiratory complex subunits Cox2, Atp4, Cyt *b* from wild-type + empty vector (lane 1), *Δmrf1Sc* + empty vector (lane 2), *Δmrf1Sc* + human mtRF1a (lane 3), and *Δmrf1Sc* + Mrf1Sc (lane 4). The right panel shows total cell extracts of the *S. pombe* Δ*mrf1* strain transformed with either the empty vector (lane 1), or plasmids producing the human mtRF1a (lane 2) or Mrf1Sp (lane 3) proteins, and analyzed by western blotting with antibodies recognizing the *S. pombe* Cox2 or human Hsp60 protein as a loading control. (D) Mitochondrial translation is restored in the presence of human mtRF1a. This was measured with ^35^S-methionine/cysteine incorporation as detailed in the Experimental Procedures. Strains were as follows: lane 1, Δ*mrf1Sc* NB345 + vector encoding human mtRF1a; lane 2, Δ*mrf1Sc* NB345 + empty vector; lane 3, WT NB346 + empty vector.

In vivo analyses were performed concomitantly. Complementation by human mtRF1a was assessed in both Δ*mrf1* yeast strains as described for mtRF1 (Figure 3). Western blots of cell fractions confirmed the mitochondrial localization of the human protein in yeast, albeit at different level to wild-type. Whereas only a faint mtRF1a signal was detected in *S. cerevisiae* mitochondria, a strong signal was obtained in *S. pombe* mitochondria, with a small fraction of protein being detected in the postmitochondrial fraction, suggesting that in *S. pombe* the human protein was either better expressed, more stable, and/or more efficiently imported into mitochondria (Figure S4A). In contrast to expression of mtRF1, the presence of human mtRF1a was sufficient to stably maintain mtDNA in the *S. cerevisiae* Δ*mrf1* strain (data not shown) and restore growth on respiratory substrates for both yeasts (glycerol for *S. cerevisiae*, Figure 3A; galactose and glycerol at 28°C for *S. pombe*, Figure 3B; and 36°C on galactose for *S. pombe*, Figure S4B), consistent with the observed increase in cytochromes (Figures 3A and 3B, right-hand panels) and partial restoration of steady-state levels of mitochondrial proteins (Cox2, Cyt *b*, Figure 3C).

Mitochondrial translation in several *S. cerevisiae* strains was also monitored by in vivo ^35^S labeling of mitochondrial proteins. No de novo synthesis was detected in the deletion mutant. In contrast, the isogenic strain producing the wild-type yeast Mrf1p exhibited high levels of protein synthesis. Production of the human mtRF1a also resulted in mitochondrial translation, albeit at decreased levels when compared to the wild-type strain (Figure 3D). These data confirm that mtRF1a can function to restore translation in yeast strains deficient in endogenous mitochondrial translation termination factors.

### Depletion of Human mtRF1a Causes a Growth Phenotype

**Figure 4:**
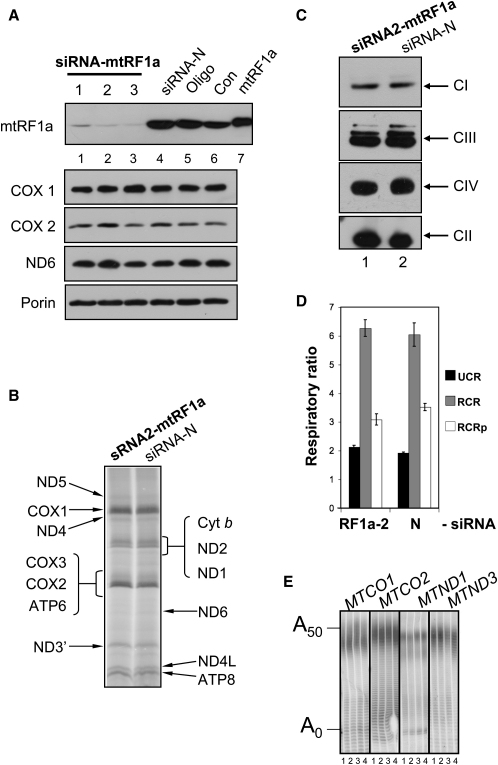
Depletion of Human mtRF1a Does Not Grossly Affect Human Mitochondrial Protein Synthesis (A) Steady-state levels of mtDNA-encoded proteins are unaffected by depletion of mtRF1a. HeLa cells were exposed to siRNA molecules targeting three different regions of the mtRF1a-encoding mRNA sequence (lanes 1–3) or to an untargeted siRNA (siRNA-N, lane 4) for 6 days, and cell lysates (30 μg) were prepared. Control lysates were made from oligofectamine alone (lane 5) and untreated cells (Con, lane 6). Western blots were performed with antibodies against mitochondrial translation products (COX1 cytochrome *c* oxidase subunit 1; COX2, cytochrome *c* oxidase subunit II; ND6 NADH CoQ oxidoreductase subunit 6). Porin was used as a nuclear encoded control to confirm equal loading. Purified mtRF1a (5 ng) is loaded in lane 7. The anti-mtRF1a experiment was overexposed to show the level of depletion. The blot accurately reflects three independent experiments. (B) De novo synthesis of mitochondrial proteins is unaffected by depletion of mtRF1a. Cytosolic protein synthesis of HeLa cells grown in the presence of siRNA targeted to *MTRF1a* (siRNA2-mtRF1a) or untargeted (siRNA-N) for 4 days was inhibited by the addition of emetine, allowing the incorporation of ^35^S-labeled met/cys directly into the 13 de novo-synthesized mitochondrial proteins. Equal amounts of total cell protein (20 μg) were separated through a 15% SDS PAG and exposed to a PhosphorImager as described. Polypeptides were designated based on their mobilities as described in Chomyn (1996). (C) OXPHOS complexes are present at normal steady-state levels in cells depleted of mtRF1a. HeLa cells were exposed to mtRF1a targeted (lane 1) or untargeted (lane 2) siRNA for 6 days prior to digitonin permeabilisation and preparation for BN-PAGE as described. Following separation, complexes were identified using antisera as detailed. Complex I, anti-39kDa antibody; complex II, anti-SDH 70 kDa antibody; complex III, anti-core 2 antibody; complex IV, anti-COX1 antibody. (D) Respiratory coupling is unaffected in cells depleted of human mtRF1a. HeLa cells (1–2 × 10^6^) grown in targeted (siRNA2-mtRF1a) and untargeted (siRNA-N) siRNA for 3 days were subjected to high-resolution respirometry as detailed. Standard errors were calculated from three measures of respiratory control and capacity. UCR compares the fully uncoupled and resting respiratory rates to give an indication of the respiratory reserve and RCR compares the fully uncoupled respiratory rates to the oligomycin inhibited state 4 respiratory rate, while RCRp combines these ratios to indicate the level of phosphorylation-related respiratory capacity. (E) The poly(A) tail of mitochondrial mRNA is unaffected by depletion of mtRF1a. RNA was isolated from HeLa cells depleted of mtRF1a (lanes 1, siRNA1; lanes 2, siRNA2; lanes 3, siRNA3) and untargeted control siRNA treated (lanes 4) before the poly(A) tail length was assessed for several mt-mRNAs as previously described (Temperley et al., 2003). Poly(A) profiles are shown for transcripts *MTCOI*, *MTCOII*, *MTND1*, and *MTND3*.

**Figure 5:**
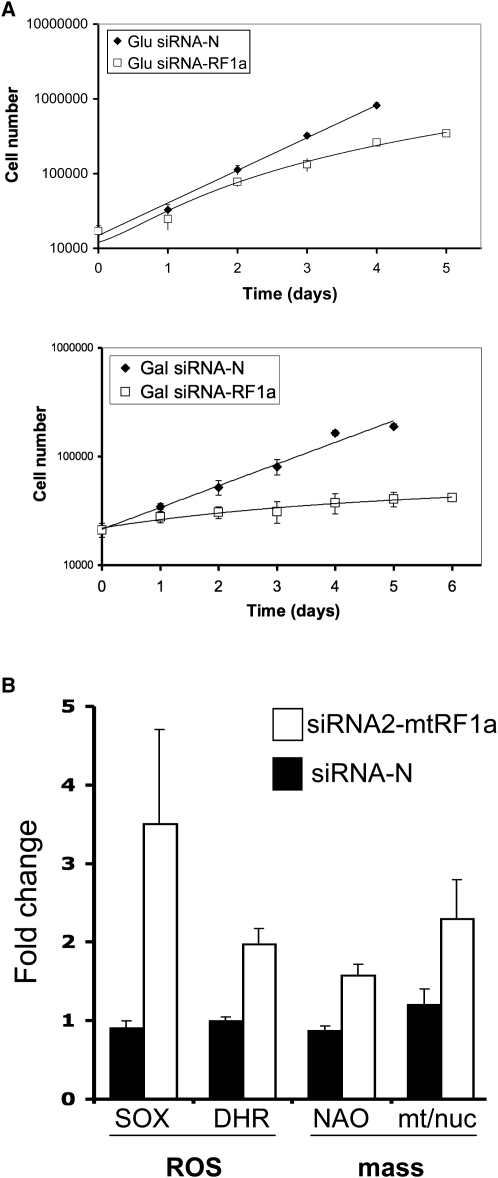
Depletion of mtRF1a Causes a Growth Defect, Increased Mitochondrial ROS Production, and Mitochondrial Mass (A) Cell growth is compromised by depletion of mtRF1a. Multiple aliquots of HeLa cells were exposed to targeted (siRNA2-mtRF1a) and nontargeted (siRNA-N) interfering RNAs for up to 6 days in glucose (promoting glycolysis) or galactose (forcing oxidative respiration) media with cell counts performed each day. Cell numbers are presented in a semi-log plot. The numbers represent a mean ± SEM from four independent wells. (B) Superoxide levels and mitochondrial mass are elevated in cells depleted of mtRF1a. HeLa cells were exposed to targeted (white) and nontargeted (black) siRNA. Superoxide (SOX) was measured with MitoSOX probes and peroxide (DHR) with dihydrorhodamine 123. Levels were measured at day 3 and compared to levels in untreated control cells. The fold increase is shown as a mean ± SEM from at least three independent experiments (p < 0.01 comparing nontargeted to targeted values for mitoSOX and DHR). Mitochondrial mass per cell was measured with the cardiolipin selective dye, NAO. Changes in mtDNA content relative to the nuclear DNA were calculated by qPCR of the *MTND1* and 18S rDNA genes and were normalized to untreated cells.

To determine the role of human mtRF1a in the mitochondrion, siRNA was used to deplete the factor in HeLa cells. Three siRNA molecules were designed to different positions of the mtRF1a transcript, and each was shown to decrease the steady-state level of the release factor by more than 75% (Figure 4A). Crude analysis of cell proliferation revealed lower levels of confluency when cells were monitored for 6 days with each siRNA independently (data not shown). As the three siRNA duplexes gave a similar phenotype, all subsequent experiments were performed with siRNA 2 alone. Detailed cell counts were then performed for siRNA2- or nontargeted siRNA-treated HeLa cells cultured in standard DMEM-promoting glycolysis or glucose-free media supplemented with galactose to force dependence on oxidative phosphorylation. As can be seen (Figure 5A), the siRNA2-treated cells showed an increase in population doubling time, which was particularly marked when cells were forced to respire in the galactose media.

### Depletion of mtRF1a Does Not Affect the Steady-State Levels of Mitochondrially Encoded RNA or Protein

To explore the exact mechanism causing this phenotype, we analyzed mitochondrially encoded RNA and protein. Figure 4 shows that the steady states of the mitochondrially encoded members of the OXPHOS complexes I (ND6) and IV (COXI and COXII) were virtually unaffected after 3 or 6 days of siRNA-mediated mtRF1a knockdown (Figure 4A). This was consistent with the absence of any effect on de novo mitochondrial protein synthesis (Figure 4B). One corollary was that nascent polypeptides remained associated with ribosomes, affecting the assembly of the nascent polypeptide into OXPHOS complexes. Thus, to assess the levels of fully assembled complexes, Blue Native PAGE was employed. After separation of intact complexes, no apparent difference was seen in the steady-state level or migration of these multisubunit enzymes (complexes I, III, and IV, Figure 4C), suggesting that nascent peptides are eventually released from the ribosome.

Although the levels of OXPHOS complexes were similar, it was possible that electron transfer rates or coupling to ATP synthesis was affected, causing the growth phenotype. Polarographic analysis of control and siRNA-treated cells was performed, and results are shown in Figure 4D. No significant differences in coupled or uncoupled rates of electron transfer were found, and the respiratory control ratios were comparable between samples, again consistent with release of the nascent and assembled polypeptide from the ribosome.

Critically, a previous report has demonstrated that loss of an mt-mRNA termination codon does not prevent protein synthesis, complex formation, or activity (Temperley et al., 2003). Thus, it appears that neither loss of the termination codon nor depletion of the release factor has an effect on the steady-state or de novo synthesis of human mitochondrial proteins. In those transcripts lacking termination codons, there was marked translation-dependent degradation of the poly(A) tail and rapid turnover (Temperley et al., 2003). Northern analysis (data not shown) and MPAT assays on *MTCO1*, *2* and *MTND1*, *3* transcripts, however, confirmed that depletion of mtRF1a caused no effect on mt-mRNA stability or poly(A) tail length (Figure 4E).

### Depletion of mtRF1a Causes Increased Mitochondrial ROS Production, Mitochondrial Mass, and mtDNA Copy Number

The role of a release factor is to promote hydrolysis of the ester bond linking the nascent peptide to the terminal tRNA. Hydrolysis can occur independent of the factor, which would potentially make the release factor redundant, but this noncatalytic rate is believed to be relatively slow (W.T., unpublished data). Moreover, peptidyl-tRNA can also “drop off” the ribosome (reviewed in Das and Varshney, 2006), and a recent report of a human mitochondrial peptidyl-tRNA hydrolase, PTH2 (De Pereda et al., 2004), suggests this enzyme would hydrolyze the peptidyl-tRNA complex, allowing the recycling of the tRNA. It is therefore difficult to predict the exact defect that would occur on depletion of the factor in human mitochondria. Our initial data showed that a growth phenotype was apparent, but this was not due to any dramatic effect on mt-mRNA stability, protein synthesis, OXPHOS complex assembly, or function. If, however, newly synthesized mitochondrial proteins are not being perfectly assembled, the activity of some OXPHOS complexes may be subtly compromised, resulting in electron escape during the natural electron transfer and generating increased reactive oxygen species. Superoxide and peroxide production in the mitochondria were therefore measured with mitoSOX and dihydrorhodamine 123 (DHR), respectively, at day 3 of siRNA-mediated mtRF1a depletion. Figure 5B shows a significant increase of these levels over control, which was maintained through to day 6 (data not shown).

Increased ROS has recently been implicated as a messenger for increased mitochondrial biogenesis (Moreno-Loshuertos et al., 2006). Levels of mitochondrial cardiolipin (quantified using NAO) and mtDNA copy number were assessed as a measure of increased mitochondrial bulk/mass. As shown in Figure 5B, a significant increase was also noted using both methods, consistent with mtRF1a depletion causing an increase in mitochondrial ROS production and mitochondrial mass. To use mtDNA copy number per cell as an indicator of mitochondrial mass, DNA was isolated from HeLa cells following exposure to the nontargeting and mtRF1a-directed siRNAs and real-time PCR employed to quantify mtDNA (ND1) relative to nuclear DNA (18S rDNA) (see the Experimental Procedures). After depletion of mtRF1a by siRNA, there was a relative 2.2-fold increase in the mtDNA levels compared to untreated controls (Figure 5B, mt/nuc), consistent with the 1.6-fold increase identified with NAO (Figure 5B, NAO).

## Discussion

We report the characterization of a human mitochondrial translation release factor, mtRF1a, based on the following criteria: (1) sequence similarity to known release factors; (2) mitochondrial localization, import, and cleavage of the preprotein; (3) presence in mitochondria of all cell types and tissues tested; (4) demonstration of release factor activity in vitro with *E. coli* ribosomes; (5) rescue of the Δ*mrf1* respiratory deficiency in fission and budding yeast in vivo; and (6) demonstration of a growth phenotype in mtRF1a-depleted human cells. Scrutiny of the primary structure of mtRF1a reveals similarities to the many other release factor sequences retaining the classic GGQ tripeptide of the peptidyl-tRNA interaction site in the M domain (Frolova et al., 1999; Song et al., 2000; Figure S2). There is greater similarity to the eubacterial RF1-type factors than to the omnipotent eRF1, consistent with this protein recognizing only UAA and UAG codons. Human mtRF1a showed no activity with UGA as codon. There are two class I release factors in most prokaryotes. Both are able to decode UAA, while UAG and UGA are recognized by only RF1 or RF2, respectively. It has previously been shown that the basis of this sequence discrimination is due to a tripeptide sequence in a structurally similar region, PXT in RF1 types or SPF for RF2 (Ito et al., 2000). MtRF1a carries PKT at position 206–208, rather than the hexapeptide found in mtRF1, consistent with this being an RF1-type release factor, terminating protein synthesis at UAA and UAG codons.

Is mtRF1 a mitochondrial release factor? The identification of mtRF1 was skillfully made through bioinformatic mining of an EST database but without any data to support either localization or function (Zhang and Spremulli, 1998). We have now shown that both mtRF1 and mtRF1a are localized to mitochondria, but only mtRF1a was capable of decoding UAA/UAG codons in our assays. Furthermore, addition of mtRF1 to release assays programmed either with mtRF1a or *E. coli* RF1 failed to compete for the ribosomal A site when UAG/UAA were present, suggesting that mtRF1 was unable to recognize these codons (Figure S5). Although for mtRF1 neither detectable in vitro release activity nor suppression of a respiratory deficiency of the yeast Δ*mrf1* was observed, we hypothesize that it is indeed a mitochondrial release factor and is responsible for decoding the noncognate stop codons AGG and AGA. The absence of release activity with the *E. coli* ribosomes may be due to the imposed use of a heterologous assay system, as the *E. coli* system does not naturally terminate with either codon. Sequence comparisons provide a compelling case for this hypothesis, since it contains the GGQ domain, has strong sequence similarity to other RF1-type proteins, and has a three residue extension immediately C-terminal to the PXT domain that is absent in all comparators (Table 1) that may underlie AGA/AGG decoding. Further, since yeast exclusively use UAA/UAG as stop codons, this could explain the lack of observed complementation in vivo.

In vivo and in vitro studies showed that mtRF1a does possess release factor activity. Expression in yeast strains devoid of their endogenous release factor restored mitochondrial translation, indicating ability to decode UAA/UAG, while depletion in human cells led to a growth phenotype and elevated ROS production. How might the loss of mtRF1a lead to increased ROS? The major source of cellular ROS is the mitochondrial electron transfer chain, in particular at the sites of electron transfer to and from respiratory complexes I and III. All 13 polypeptides encoded by the human mtDNA are members of four of the five complexes that couple oxidative phosphorylation. These complexes contain many polypeptides imported from the cytosol and integrated together with the mitochondrially encoded proteins in a complicated and incompletely understood process of assembly. The 13 mitochondrial polypeptides are highly hydrophobic, being putatively translated at the membrane surface and nursed by the membrane protein Oxa1 that aids in the assembly process (Liu and Spremulli, 2000). In the absence of translation termination, nascent proteins will be inserted into the membrane but will also still be attached to the ribosome via the P site bound peptidyl-tRNA. It is understood that this ester bond can be spontaneously hydrolyzed over time, but release factor activity at the ribosome dramatically accelerates this natural process (Schmeing et al., 2005). However, it is possible that subtle temporal effects on the assembly process following siRNA-mediated knockdown may lead to assembled complexes with minor defects in electron transfer, leading to increased loss of electrons in the form of ROS. A similar situation has recently been reported for *trans*-mitochondrial murine cybrids, in which the same mtDNA polymorphism in different nuclear backgrounds led to modulations in ROS production and mitochondrial cell mass without any dramatic effect on OXPHOS complex activities (Moreno-Loshuertos et al., 2006). A second possibility, that we have discounted, is that the ribosome might stall at the termination site and in the absence of a release factor shift-reading frame, leading to C-terminal extensions mainly of polylysine due to the proximity of the poly(A) tail. First, in the only characterization of a human mt-mRNA lacking a termination codon, the mt-mRNA was rapidly degraded following translation, probably due to the translating ribosome pushing away the natural poly(A)-associated protein(s) leaving naked RNA prone to RNase digestion (Temperley et al., 2003). In the study presented here, there is no evidence of decreased mt-mRNA stability. Second, although only short polylysine extensions would be predicted to be added to most mitochondrial polypeptides, ATPase 8, in contrast, would increase substantially in size, as it is translated from the 5′ part of the bicistronic *RNA14*. Following depletion of mtRF1a, there is no evidence of increased size in this or any newly synthesized mitochondrial proteins. Third, when we have used an antibody specific to polylysine, we do not see any new mitochondrial products (data not shown).

In summary, we report the identification and biochemical characterization of a human mitochondrial translation release factor, mtRF1a, that decodes the major stop codons UAA and UAG. This should now be included in the growing list of mitochondrial translation factors, mutations of which could underlie the mitochondrial dysfunction in an increasing number of patients for whom nuclear-encoded mutations have yet to be determined.

## Experimental Procedures

### Production of GFP and GST Fusion Constructs and Cloning into Yeast Expression Vectors

Exact details of primers and construct design are given in the Supplemental Experimental Procedures.

### Yeast Growth Conditions, Plasmid and General Strain Constructions, and Complementation Assays

All yeast strains used in this study are detailed in Table S1.

Yeast media, general genetic techniques, and transformation protocols were as described in Chiron et al. (2005). Briefly, whereas media containing 2% glucose was fermentable for both yeasts, nonfermentable conditions were achieved in media containing as sole carbon source 2% glycerol (*S. cerevisiae*), or for *S. pombe* 3% glycerol/ethanol or 2% galactose and 0.1% glucose.

### Production of an *S. pombe* Δmrf1 Strain

A single *S. pombe* protein, encoded by gene Spac2f7.17, was found to have a high level of identity (65%) with both *E. coli* RF1 and *S. cerevisiae* Mrf1. The ORF was PCR amplified using a cDNA library as template and cloned into the BamHI site of the pTG1754 expression plasmid. An *S. pombe Δmrf1::Kan^R^* strain (NB329) was constructed in the wild-type recipient strain NB205-6A (Chiron et al., 2005) by using a PCR disruption strategy with hybrid *mrf1-Kan^R^* oligonucleotides as described in (Chiron et al., 2005). Clones showing delayed growth on galactose medium were proved by PCR to contain the correct insertion. Crossing to a wild-type followed by sporulation and tetrad analysis showed a 2:2 cosegregation of the geneticin resistance and slow galactose growth. Transformation with the *mrf1Sp* plasmid restored growth on galactose, showing that the mitochondrial DNA was intact in this strain.

### Spectroscopic Analyses

Cytochrome spectra were recorded from transformants of both yeasts grown on solid medium. Cells were harvested, dried, and mixed with sodium dithionite to fully reduce the cytochromes and were frozen in liquid nitrogen before recording the absorbance of the samples at wavelengths from 630 to 490 nm (spectrophotometer Cary 400). Peaks were the following, for *S. cerevisiae* or *S. pombe*, respectively: cytochrome *c* (546 or 548 nm), cytochrome *c_1_* (552 or 554 nm), cytochrome *b* (558 or 560 nm), and cytochrome *aa_3_* (602 or 603 nm).

### Overexpression and Purification of Human Mitochondrial and Bacterial Proteins

*E. coli* strain Rosetta(DE3)pLysS (Novagen) was transformed with constructs for the overexpression of the human mitochondrial RFs that were purified as detailed in the Supplemental Experimental Procedures. *E. coli* RF1 was overexpressed and purified from strain BL21 pLysS following the protocol described in Tate and Caskey (1990).

### Mitochondrial Import Assays

Reactions (20 μl) containing 10 μl rabbit reticulocyte lysate (Promega), 35 μCi EasyTag express protein labeling mix (NEG-772, PerkinElmer), and 17.5 μM amino acids mix lacking methionine (Promega) were programmed with 5 μg of in vitro-transcribed RNA (synthesized using AmpliScribe, Epicenter) for 1 hr at 30°C, after which 5 mM cold methionine and 250 mM sucrose were added followed by a further 15 min incubation before ultracentrifugation at 125 kg for 30 min at 4°C.

Rat liver mitochondria were isolated as previously described (McGregor et al., 2001). Each 100 μl import reaction contained 200 μg of isolated mitochondria and 5 μl of the in vitro-translated protein in isolation buffer (220 mM Mannitol, 70 mM sucrose, 10 mM HEPES [pH 7.4], 1 mM MgCl_2_, 1 mM EGTA) supplemented with 1 mM ATP, 1 mM NADH, 20 mM succinate, and 50 μM CCCP as indicated. Reactions were incubated for 1 hr at 30°C, after which 5 μg Proteinase K was added to a subset of reactions followed by a further 30 min incubation on ice. PMSF (1 mM) was added to each reaction prior to centrifugation at 13 kg for 1 min at 4°C. Pellets were washed in isolation buffer, repelleted, and resuspended in dissociation buffer prior to separation by SDS PAGE. Visualization was effected by PhosphorImage analysis with ImageQuant software (Molecular Dynamics, GE Healthcare).

### Cell Preparations and Western Analysis

Details of all yeast and human cell preparations and protein analysis techniques are given in the Supplemental Experimental Procedures.

### In Vitro Translation Termination Assay

This was performed essentially as described (Caskey et al., 1971; Tate and Caskey, 1990) with modifications detailed in the Supplemental Experimental Procedures. To generate the f[3H]met-tRNAmet, the following were combined to give the final concentrations indicated: cacodylate buffer (100 mM [pH 6.8]), 3.5 mM leucovorin (Sigma) as the formyl donor, 20 μM amino acids (Promega), 0.3 mg N-formylmethionine-specific tRNA (Sigma), 1.2 mM ATP, 1 mM DTT, 10 mM MgCl2, 3.8 nmol L-[*methyl*-3H] methionine (GE Healthcare), and cold methionine up to 21.8 nmol, with the aminoacyl tRNA synthetase. This was incubated for 30 min at 37°C. Ribosomal substrate (amounts given are per 50 μl assay) was prepared by incubation of 70S ribosomes (5 pmol) with AUG (250 pmol) and f[^3^H]met-tRNA^met^ (2.5 pmol) in 20 mM Tris-HCl (pH 7.4), 10 mM Mg(OAc)_2,_150 mM NH_4_Cl at 30°C for 20 min. This “activated” ribosomal substrate was stored on ice prior to interrogation with release factors for activities with selected codons. Standard release reactions were as described in the Supplemental Experimental Procedures, with f[^3^H]met in the supernatant quantified as an indicator of release activity.

### siRNA Transfection

siRNA transfection was performed using standard methods as detailed in the Supplemental Experimental Procedures.

### Free Radical and Mitochondrial Mass Measurements

MitoSOX (Molecular Probes) was prepared in DMSO and diluted to 5 μM in DMEM medium lacking serum. Cells were then incubated for 10 min at 37°C, washed before resuspension in 2 ml DMEM-lacking serum, and passed through a Partec PAS flow cytometer (Münster, Germany). Forward and side scatter data were collected, as was autofluorescence of unstained cells. Gating then allowed elimination of debris and apoptotic cells from the analyses. Red fluorescence emission resulting from MitoSOX staining was detected and median fluorescence intensity analyzed using FloMax software. The flow cytometer was calibrated using fluorescent microspheres. Each measurement was performed in triplicate based on 10,000 events and the experiment repeated on four independent occasions.

Dihydrorhodamine 123 (DHR, Molecular Probes) or nonyl acridine orange (NAO, Molecular Probes) was resuspended in DMSO, diluted to 30 μM (DHR) or 10 μM (NAO) in DMEM-lacking serum, added to cells, and incubated at 37°C for 30 (DHR) or 10 (NAO) min. Cells were then washed before resuspension in 2 ml DMEM-lacking serum and the green fluorescence emission analyzed as above.

### Relative Quantification of mtDNA Copy Number

Real-time PCR was performed on DNA isolated from cells digested with Proteinase K (2 mg/ml final) in TE buffer (pH 7.4), 1% SDS that was extracted with phenol and ethanol precipitated.

PCR primer and fluorogenic FAM probes for regions of mitochondrial *ND1* (forward primer, L3485–3504; reverse primer, H3532–3553; probe, L3506–3529; Anderson et al. [1981]) and nuclear 18S (forward primer, 1050–1071; reverse primer, 1095–1115; probe, 1074–1093, accession number M10098) genes were designed using Primer Express software (Applied Biosystems). Each 25 μl reaction was performed in duplicate on three independent samples. To each DNA sample (10 ng) was added 12.5 μl TaqMan Universal PCR MasterMix (Applied Biosystems), primers (300 nM final each), fluorogenic probes (100 nM final), and H_2_O. PCR and fluorescence analysis was performed using the Perkin Elmer GeneAmp 5700 sequence detection system. The amplification profile was 50°C, 2 min; 95°C, 10 min; 40 cycles of 95°C, 15 s, and 60°C, 1 min. Threshold cycle number (Ct) was calculated with GeneAmp 5700 SDS software v1.7 (Applied Biosystems).

*S. pombe* mitochondrial DNA was extracted from transformed cells grown in minimal medium lacking uracil at 28°C or 36°C and analyzed by Southern blotting with either the 1.3 kb *arg1Sp* nuclear gene ORF (accession SPCC777.09c) or the pDG3 plasmid containing the full *S. pombe* mitochondrial DNA cloned on pBR322 as described in Chiron et al. (2005).

### De Novo Mitochondrial Protein Synthesis

Protein translation in human cells was performed essentially as described (Chomyn, 1996) and detailed in the Supplemental Experimental Procedures.

### Determination of mRNA Poly(A) Tail Length

This method was described in detail in Temperley et al. (2003). All relevant primers used in this study are given in the Supplemental Experimental Procedures.

### Respirometry

High-resolution respirometry was performed at 37°C using an Oroboros Oxygraph-2K (Oroboros Instruments, Innsbruck, Austria) essentially as detailed in Hutter et al. (2004). Approximately 0.5–1 × 10^6^ cells were added to the chamber in 2 ml of respiration buffer (glucose free DMEM, 10% [v:v] FCS, 0.11 mg/ml-1 Na pyruvate, 0.9 mg/ml galactose), and routine respiratory oxygen flux (R) was established over 15 min with automatic deduction of background flux using the online DatLab software (Oroboros Instruments, Innsbruck, Austria). ATP synthase was then inhibited (oligomycin, 1 μg/ml) and the rate measured (R_o_). Fully uncoupled flux (R_u_) was then determined by sequential titration with 0.5 μM boluses of FCCP until maximal rates were observed. Respiration was fully inhibited by the addition of rotenone (complex I inhibitor, 0.5 μM final) and antimycin A (complex III inhibitor, 2.5 μM). Where necessary, sporadic reaerations were performed to avoid oxygen limitation during these measurements, cell counts were made using a small aliquot of cells at the end of the experiment, and rates were then normalized to pmol oxygen consumed per 10^6^ cells per unit time. To assess the relative coupling, three ratios were calculated: the respiratory control ratio (RCR, õ_Ru_/õ_Ro_), the uncoupling control ratio (UCR, õ_Ru_/õ_R_) as a measure of respiratory reserve capacity, and a combination of these two ratios (RCRp, õ_R −_õ_Ro_/õ_Ru_) to determine the proportion of phosphorylation-related respiratory capacity.

### Statistical Analysis

Where required, Student's unpaired t tests were used to determine the significance of values as indicated in figure legends. Values of p < 0.05 were recorded as statistically significant.

## Figures and Tables

**Table 1:** Comparison of the Primary Sequence Covering the Stop Codon Recognition Motif of RF1-Type Release Factors from Various Organisms

Species	Sequence																					Identity	Accession Number	Cytosolic/Mitochondrial
*E. coli*	H	R	V	Q	R	V	P	A	T	-	-	-	E	S	Q	G	R	I	H	T	S	18/18	P07011	Cytosolic
Human	H	R	V	Q	R	I	P	E	V	G	L	S	S	R	M	Q	R	I	H	T	G	10/21	BC042196	Mitochondrial
Human	H	R	V	Q	R	V	P	K	T	-	-	-	E	K	Q	G	R	V	H	T	S	15/18	BC011873	Mitochondrial
*Streptomyces coelicolor*	H	R	V	Q	R	V	P	A	T	-	-	-	E	S	Q	G	R	I	H	T	S	18/18	CAB94531	Cytosolic
*Paracoccus denitrificans*	H	R	V	Q	R	V	P	E	T	-	-	-	E	S	G	G	R	I	H	T	S	16/18	YP_914701	Cytosolic
*Bacillus subtilis*	H	R	V	Q	R	V	P	E	T	-	-	-	E	S	G	G	R	I	H	T	S	16/18	CAA89884	Cytosolic
*Rickettsia prowazekii*	H	R	V	Q	R	I	P	E	T	-	-	-	E	S	Q	G	R	I	H	T	S	16/18	Q9ZD21	Cytosolic
*Kluyveromyces lactis*	H	R	V	Q	R	V	P	A	T	-	-	-	E	S	K	G	R	T	H	T	S	16/18	P41767	Mitochondrial
*Saccharomyces cerevisiae*	H	R	V	Q	R	I	P	S	T	-	-	-	E	T	K	G	R	T	H	T	S	15/18	P30775	Mitochondrial
*Schizosaccharomyces pombe*	H	R	V	Q	R	T	P	A	T	-	-	-	E	T	K	G	R	V	H	T	S	14/18	CAA90504	Mitochondrial
*Caenorhabditis elegans*	H	R	V	Q	R	V	P	V	N	-	-	-	-	-	D	S	R	M	H	T	S	11/18	O44568	Mitochondrial

## Supplemental Data

Document S1. Supplemental Experimental Procedures, Supplemental References, One Table, and Five Figures
