# Supplementary material for: mtRF1a Is a Human Mitochondrial Translation Release Factor Decoding the Major Termination Codons UAA and UAG
Source: Mol Cell. 2007 Sep 7;27(5):745–57. doi: 10.1016/j.molcel.2007.06.031 (PMC1976341; doi:10.1016/j.molcel.2007.06.031)

Molecular Cell, Volume *27*

Supplemental Data

mtRF1a Is a Human Mitochondrial Translation Release Factor Decoding the Major Termination Codons UAA and UAG

Hamid Reza Soleimanpour-Lichaei, Inge Kühl, Mauricette Gaisne, Joao F. Passos, Mateusz Wydro, Joanna Rorbach, Richard Temperley, Nathalie Bonnefoy, Warren Tate, Robert Lightowlers, Zofia Chrzanowska-Lightowlers

Supplemental Experimental Procedures

Detailed experimental procedures

Constructs containing putative human mitochondrial release factors

Clones for the 2 putative mitochondrial release factors were obtained from MGC; mtRF1 (IMAGE id 5198308, accession number BC042196) and mtRF1a (3029407, accession number BC011873). These were used as templates in PCR amplifications to generate the constructs below.

Production of GFP- and GST-fusion constructs and cloning into yeast expression vectors

All primers used below included *Bam*H1 or *Not* 1 cleavage sites (underlined). An amplicon incorporating nt 89 to 972 of the cDNA encoding mtRF1 was formed using primers RF1-GFPfor - 5'-CTCTCT<u>GGATCC</u>TTAGATGCTGAGATGAATCG-3' and RF1-GFPrev 5'-CTCTCT<u>GGATCC</u>TCATCTGGCTGAGGAAG-3' in a standard PCR reaction with a 55˚C-annealing temperature. Similarly, an amplicon incorporating nt 12 to 1099 of the cDNA encoding mtRF1a was PCR generated using primers RF1a-GFPfor - 5'CTCTCT<u>GGATCC</u>GATCTCGGACTAAGGATG-3' and RF1a-GFPrev 5'-CTCTCT<u>GGATCC</u>GCATCCAGTAGATAATCTCC-3' in a standard reaction with a 53˚C annealing temperature. Amplicons were digested and cloned in-frame upstream of GFP in pGFP3 (kindly donated by Dr D. Elliott, Newcastle), resulting in the N-terminal fusion of 290 and 357 residues of mtRF1 and mtRF1a to GFP, respectively.

To produce the glutathione-S-transferase (GST)-fusion constructs, amplicons incorporating nt 249 to 1511 of cDNA encoding mtRF1 were generated with primers 1-

GSTfor 5'-TCTCTCGGATCCCTTCATCTGTTAAGTAAGAATTGG-3' and 1-GSTrev 5'-TCTCTCGGATCCAAGCTTCAGTCTGCCTC-3' (59˚C annealing), and mtRF1a (nt 124 to 1534) was amplified with primers 1a-GSTfor 5'-TCTCTCGGATCCCTGGAGGAGCTGTTCG-3' and 1a-GSTrev 5'-TCTCTCGGATCCTGAGGAGCTCCTTAGG-3' (52˚C). Amplicons were digested and fused in-frame downstream of GST to facilitate purification.

Finally cDNAs containing the entire ORFs were amplified using *Not*1-1for 5'-CTCTCTGCGGCCGCTGCTGAGATGAATCGTCAC-3'/Not1-1rev 5'-CTCTCTGCGGCCGCGATTTCCAAGCTTCAGTCTG-3' or Not1-1afor 5'-CTCTCTGCGGCCGCATCTCGGACTAAGGATGC-3'/Not1-1arev 5'-CTCTCTGCGGCCGCAGAGTAAGGGTACTTTCACC-3' primer pairs for mtRF1 and mtRF1a respectively. *Not*I sites are underlined and restriction fragments from both cDNAs were subcloned in forward orientation into the *Not*I sites of the *S. cerevisiae URA3* expression vector pFL61 (Minet et al., 1992) and of the *S. pombe ura4* expression vector pTG1754 (a gift from Transgene, (Chiron et al., 2005)). Both vectors contain strong constitutive promoters.

Yeast growth conditions, plasmid and general strain constructions and complementation assays

The *S. cerevisiae MRF1* ORF was PCR amplified from genomic DNA and cloned into pFL61-BEB (a derivative of pFL61 containing a *Bam*HI site). The *S. cerevisiae MAT*α Δ*mrf1::Kan*^{R} haploid strain Y14510 was ordered from Euroscarf, depleted of mtDNA by growth on ethidium bromide containing medium, and crossed to a wild type strain containing an intron-less mitochondrial genome (CW252/A, a *MATa* version of CW252, (Saint-Georges et al., 2002). The resulting heterozygous Δ*mrf1* diploid KV2 and a control wild type diploid were transformed with the control vector or plasmids producing the human or yeast proteins. Diploids were sporulated and tetrads dissected (Sanger micromanipulator) on minimal medium lacking uracil to maintain the plasmids. For biochemical studies isogenic strains were generated by transforming the *rho°* versions of Y14510 (Δ*mrf1Sc*) or BY4742 (wt) with empty vector or one producing either *S. cerevisiae* Mrf1 or human mtRF1a, and then introducing the intron-less mtDNA through cytoduction with the karyogamy deficient strain JC8/252.

Tissue culture

Human HeLa, HepG2 and HEK293 cells were cultured (37˚C, humidified 5% CO_2) in Eagle's modified essential medium (Sigma) supplemented with 10% (v/v) foetal calf serum, non-essential amino acids and 2 mM L-glutamine. For growth on respiratory substrates cells were cultured in glucose free DMEM (Gibco) supplemented with 0.9 mg/ml galactose, 1 mM sodium pyruvate, 10% (v/v) foetal calf serum, non-essential amino acids and 2 mM L-glutamine.

Transient transfection of HeLa cells, microscopy and image capture

HeLa cells were grown to 50% confluency on coverslips and transfected with vectors expressing the GFP fusion constructs (1 µg) using Superfect (Qiagen) as recommended. Cells were cultured for a further 24 h prior to incubation with Mitotracker Red CM-H2XRos (1 µM final, Invitrogen). After brief fixation (4% paraformaldehyde in PBS, 15 min at room temperature), cells were mounted in Vectashield (H-1500 Vector Laboratories Inc) and visualised by fluorescence microscopy using a Leica (Nussloch Germany) DMRA. Images were recorded as a Z-series (0.5 µm slices) using a cooled CCD camera and imaging system (Spot –II Diagnostics Instruments, Sterling Heights MI, USA).

Over-expression and purification of human mitochondrial and bacterial proteins

E.coli strain Rosetta(DE3)pLysS (Novagen) was transformed with constructs for the overexpression of the human mitochondrial release factors. IPTG (1mM) was added to bacterial cultures (0.40-0.45 A_{600nm}) and induced for 16 h at 16°C. Cells were harvested, resuspended in PBS and sonicated 10 × 10 s, amplitude 18 microns (Soniprep 150). The lysate was centrifuged at 35,000x*g* for 20 min and the supernatant filtered before application to a Glutathione Sepharose 4B (Amersham Bioscience) column. The column was washed with PBS/150mM NaCl and protein released from GST by cleavage with PreScission Protease (Amersham Bioscience), resulting in the removal of 49 N-terminal residues for purified mtRF1 and 32 residues for mtRF1a. *E.coli* RF1 was overexpressed and purified from strain BL21 pLysS following the protocol described in (Tate and Caskey, 1990).

***In vitro* translation termination assay**

This was performed essentially as described in (Caskey et al., 1971; Tate and Caskey, 1990) with complexes simulating the state of the ribosome at the terminal stage of protein synthesis being generated to test release activity of proteins with specific RNA oligomers.

Modifications to the published protocol included increasing the concentrations of putative stop codons from 250 to 400 pmol, release factor from 5 to 50 pmol, and time of incubation to 40 min.
70S ribosomes, amino-acyl tRNA synthetase and formylating activities were all isolated from purchased MRE 600 paste (UAB Fermentation facility, Birmingham, Alabama USA). All RNA oligomers were custom synthesised by Dharmacon. No modifications were made to the protocol for the generation of the f[^{3}H]met-tRNA^{met} substrate. The following were combined in cacodylate buffer (100 mM pH 6.8), 3.8 nmol L-[*methyl*-^{3}H] methionine (GE Healthcare) and cold methionine up to 21.8 nmol, 3.5 mM leucovorin (Sigma) as the formyl donor, 20 µM amino acids (Promega), 0.3 mg N-formylmethionine specific transfer ribonucleic acid (Sigma), 1.2 mM ATP, 1 mM DTT, 10 mM $MgCl_2$ and incubated for 30 min at 37˚C. The reaction was extracted with phenol, the tRNA precipitated with 95% ethanol and the pellet washed 4 times in ethanol. The final pellet was resuspended in H_2O and stored at -80˚C.

Ribosomal substrate (amounts given are per 50 µl assay) was prepared by incubation of 70S ribosomes (5 pmol) with AUG (250 pmol), and f[^{3}H]met-tRNAmet substrate (2.5 pmol) in 20 mM Tris-HCl pH7.4, 10 mM $Mg(OAc)_{2,}$150 mM NH_4Cl at 30˚C for 20 min. This 'activated' ribosomal substrate was stored on ice prior to interrogation with release factors for activities with selected codons. Standard release reactions were carried out with the activated ribosomes as described, RNA oligomers, release factor (amounts as in figure legends) in 50 mM Tris-HCl pH7.2, 30 mM $Mg(OAc)_{2,}$ 75 mM NH_4Cl, 10% ethanol, incubated at 24˚C for 40 min. Reactions were quenched by the addition of HCl (83mM final) and ethyl acetate extracted. Tritiated methionine in the supernatant was quantified as an indicator of release activity.

Cell preparations and western analysis

Lysate was prepared by pelleting human cells from 75 cm^2 flasks and resuspending in 150 µl of lysis buffer (50mM Tris-HCl pH 7.5, 130mM NaCl, 2mM $MgCl_2$, 1% Nonidet P-40, 1 mM PMSF and Roche EDTA protease inhibitor cocktail). This was vortexed briefly, spun at 1500*g* for 5 min at 4˚C and the supernatant retained.

Human mitochondrial fractions were prepared from cell pellets resuspended in 0.6 M mannitol, 10 mM Tris- HCl pH7.4, 1 mM EGTA, 0.1% BSA and hand homogenised by 15 passes in a Teflon:glass tissue grinder. Following centrifugation at 600*g* the supernatant was retained and the pellet was re-homogenised and spun as before. The combined supernatants were cleared at 600*g* and then the crude mitochondria pelleted at

15k*g*. These were washed in buffer lacking BSA and frozen in liquid nitrogen until required. *S. cerevisiae* and *S. pombe* mitochondria were purified from cells grown in minimal galactose (*S. cerevisiae*) or glucose (*S. pombe*) media lacking uracil as described in (Wallis et al., 1994) and (Chiron *et al*., 2007) respectively, except that protease inhibitors tablets (Roche) were added before breaking the protoplast and during all subsequent steps. *S. pombe* total proteins were extracted as in (Chiron et al. 2005).

Human proteins were separated by SDS–PAGE and immobilized by wet transfer (100 V, 2.5 h at 4˚C) on to PVDF (Immobilon-P, Millipore Corporation) membrane in 25 mM Tris, 192 mM glycine, 0.02% (w/v) SDS and 15% (v/v) methanol. Proteins of interest were bound by overnight incubation (4˚C) with rabbit polyclonal antibodies [mtRF1a, Porin, COX I, COX II, SDH 70K and ND6 (MitoSciences)] followed by HRP-conjugated secondary antibodies (DAKO) and visualized by ECL-plus (GE Healthcare). Antisera to human mtRF1 and mtRF1a were generated by Eurogentec using recombinant proteins as antigens. Yeast proteins were immobilized onto PVDF or nitrocellulose (Schleicher & Schull) by wet or dry transfer in 25 mM Tris, 192 mM glycine, 0.02% (w/v) SDS and 20% (v/v) ethanol. Blots were hybridized with mouse [anti-Cox2Sc, -Porin, -PGK, all Invitrogen] or anti-human HSP60 [a kind gift from P.Belengeur] or rabbit antibodies [anti-mtRF1a; -Cox2Sp, (Gaisne and Bonnefoy, 2006); -Arg8Sc (Steele et al., 1996) ; -Cyt *b*, a gift from C. Lemaire; -Atp4, a gift of Jean Velours).

Blue native gel electrophoresis was performed as described in (Nijtmans et al., 2002) with the modification that 30 µg protein was loaded per lane and wet transfer as above.

siRNA transfection

Both negative control and mtRF1a specific siRNA duplexes were purchased pre-annealed from Eurogentec. The negative control is a unique sequence that does not match any sequence in the genome. Sense strands for siRNA specific to the mRNA encoding mtRF1a were :- 1) GACGCUGCAUGAUCUUGAAdTdT, 2) CCAUGACUGUAGCAAUAUUdTdT and 3) CGAUGAGAAUGAAGAUUUAdTdT.

HeLa cells in a 35 mm dishes were grown to 20-30% confluency, incubated in 800 µl DMEM (D6429, Sigma) supplemented with 10% FCS, 50 µg/ml uridine and 1x non-essential amino acid, to which the Oligofectamine (Invitrogen) transfection mix in OptiMEM (Gibco) was added to give a final concentration of 0.2 µM siRNA. Cells were cultured in this medium for 3 days and then either harvested or retransfected for the 6 day time point.

De Novo mitochondrial protein synthesis

HeLa cells were cultured in 35 mm dishes until 70% confluent, then incubated in methionine free medium for 1 h. This medium was replaced with 1 ml labeling medium (methionine/cysteine-free DMEM, 10% dialyzed FBS, emetine 10 µg /ml, and 500 µCi [^{35}S]-methionine/cysteine [3,000 Ci/mmol; Amersham]) and cells incubated at 37°C for 2 h. After removal of the incubation medium, the cells were washed in PBS and pelleted. Protein samples (20 µg) were separated on 12% SDS PAGE and products visualized with a PhosphorImager system with ImageQuant software (Molecular Dynamics, GE Heathcare). *S. cerevisiae* transformants were grown in minimal galactose medium lacking uracil, harvested at the exponential phase, resuspended in 40 mM KH_2PO_4/ K_2HPO_4, 2% galactose buffer pH 6 and incubated for 1 h at 28°C. After a 10 min pre-incubation with 600 µg/ml cycloheximide to block cytosolic translation, 72 µCi of [^{35}S] methionine/cysteine (Redivue Pro-mix, Amersham) were added and incubation was carried out for 30 min. After removal of the medium a 10 min chase was performed by adding 12 µl of 0.25 M methionine and 0.25 M cysteine. Equal amount of total proteins samples were loaded on a 16% acrylamide-0,5% bisacrylamide gel and products visualized by autoradiography or PhosphorImaging as above.

Determination of mRNA poly(A) tail length (MPAT)

The additional primers used in this paper to analyse the following transcripts were as follows :- *MTCO1*, first primer 5'-CATATTCATCGGCGTAAATC-3', nested radiolabelled primer 5'-CAACCCCATGGCCTCCA-3' ; *MTCO2*, first primer 5'-GGTATACTACGGTCAATG-3', nested radiolabelled primer 5'-TATAGCACCCCCTCTACCCC-3' ; *ND1* first primer 5'-TAAACACCCTCACCACTACA-3', nested radiolabelled primer 5'-TACAATCTCCAGCATTCCC-3' ; *ND3* first primer 5'-CCGCCCGCGTCCCTTTC-3', nested radiolabelled primer 5'-GACTACAAAAAGGATTAGACT-3'. An annealing temperature of 54˚C was used for all reactions except with *MTCO2* first primer, which was 50˚C.

Supplemental References

Minet, M., Dufour, M. E., and Lacroute, F. (1992). Cloning and sequencing of a human cDNA coding for dihydroorotate dehydrogenase by complementation of the corresponding yeast mutant. Gene *121*, 393-396.

Nijtmans, L., Henderson, N., and Holt, I. (2002). Blue Native electrophoresis to study mitochondrial and other protein complexes. Methods *26*, 327-334.

Saint-Georges, Y., Bonnefoy, N., di Rago, J. P., Chiron, S., and Dujardin, G. (2002). A pathogenic cytochrome b mutation reveals new interactions between subunits of the mitochondrial bc1 complex. J Biol Chem *277*, 49397-49402

Steele, D. F., Butler, C. A., and Fox, T. D. (1996). Expression of a recoded nuclear gene inserted into yeast mitochondrial DNA is limited by mRNA-specific translational activation. Proc Natl Acad Sci U S A *93*, 5253-5257.

Wallis, M. G., Groudinsky, O., Slonimski, P. P., and Dujardin, G. (1994). The NAM1 protein (NAM1p), which is selectively required for cox1, cytb and atp6 transcript processing/stabilisation, is located in the yeast mitochondrial matrix. Eur J Biochem *222*, 27-32.

Table S1

Name	Nuclear genotype	Mt genotype	Source
S. cerevisiae			
Y14510	*MATα his3Δ1 leu2Δ0 lys2Δ0 ura3Δ0*	*$rho^{-/0}$ [introns?]* [1]	Euroscarf
Y14510rho^0	*MATα his3Δ1 leu2Δ0 lys2Δ0 ura3Δ0*	*rho^0*	This work
BY4742	*MATα his3Δ1 leu2Δ0 lys2Δ0 ura3Δ0 mrf1Sc::KanMX4*	*rho^+ [introns?]* [1]	Euroscarf
BY4742rho^0	*MATα his3Δ1 leu2Δ0 lys2Δ0 ura3Δ0 mrf1Sc::KanMX4*	*rho^0*	This work
CW252/A	*MATa ade2-1 his3-11,15 trp1-1 leu2-3,112 ura3-1 can1-100*	*rho^+ [Δi]*	G. Dujardin
KV2	*MATα/a ade2-1/+ his3-11,15/his3Δ1 trp1-1/+ leu2-3,112/leu2Δ0 lys2Δ0/+ ura3-1/ura3Δ0 mrf1Sc::KanMX4/+ can1-100/+*	*rho^+ [Δi]*	This work
JC8/252	*MATa leu1 can^R*	*rho^+ [Δi]*	G. Dujardin
NB345 + pFL61	*MATα his3Δ1 leu2Δ0 lys2Δ0 ura3Δ0 mrf1Sc::KanMX4*	*$rho^{-/0}$ [Δi]* [2]	This work
NB345 + pMRF1Sc or pMTRF1a	*MATα his3Δ1 leu2Δ0 lys2Δ0 ura3Δ0 mrf1Sc::KanMX4*	*rho^+ [Δi]* [2]	This work
NB346	*MATα his3Δ1 leu2Δ0 lys2Δ0 ura3Δ0*	*rho^+ [Δi]*	This work
S. pombe			
NB205-6A	*h- ade6-M216 his3Δ leu1-32 ura4-D18*	*rho^+*	Chiron *et al.,* 2005
NB329	*h- ade6-M216 his3Δ leu1-32 ura4-D18 mrf1Sp::KanMX4*	*rho^+*	This work

[Δi]: intron-less genome with wild-type cytochrome *b* (Saint-Georges *et al*., 2002); **[1]** Mitochondrial intron content unknown; **[2]** NB345 remains *rho^+* when transformed with pFL61 carrying the genes coding Mrf1Sc (pMRF1Sc) or HmtMRF1a (pMTRF1a), and becomes $rho^{-/0}$ when transformed with the empty pFL61 vector.

Figure S1. Southern blot analysis of the mitochondrial DNA profile in the Δ*mrf1Sp* strain. Total genomic DNAs were digested with *Hin*DIII. The blot in the left panel (28°C) was probed first with the nuclear *arg1Sp* and reprobed with the full mitochondrial DNA in a plasmid. The latter also hybridizes weakly with the pTG1754 plasmid backbone. In the right hand blot from cells grown at 36° C, the two probes were mixed in a single hybridization. Strains were the following for both temperatures, from left to right: NB205-6A (wt) + empty vector; NB329 (Δ*mrf1Sp*) with empty vector; or with the vector producing HmtRF1a; or Mrf1Sp.

Supp Mat Fig 1

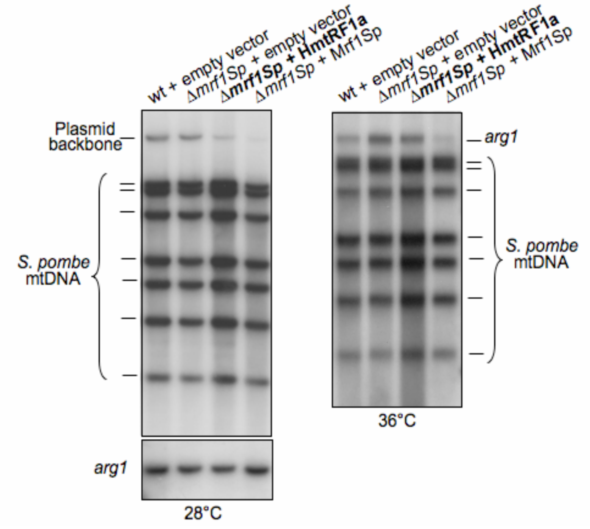

Figure S2. Alignment of human mitochondrial mtRF1 and mtRF1a with selected release factors. (**A**) The 2 human sequences (1, 2) were aligned with those from *E. coli* RF1 (3), another bacterial RF1 (4), its putative ancestor *Rickettsia prowazekii* (5), and the mitochondrial RFs from yeast (6, 7, 8) and nematode (9). Relative similarities are indicated by the blocks beneath. Boxes indicate the GGQ and the PXT domains. The latter encompasses the 18 amino acid region detailed in Table 1. (**B**) The 2 human sequences are aligned to show both overall levels of identity and similarity, and specifically within the boxed regions of the anticodon PXT motif and α5-helix tip.

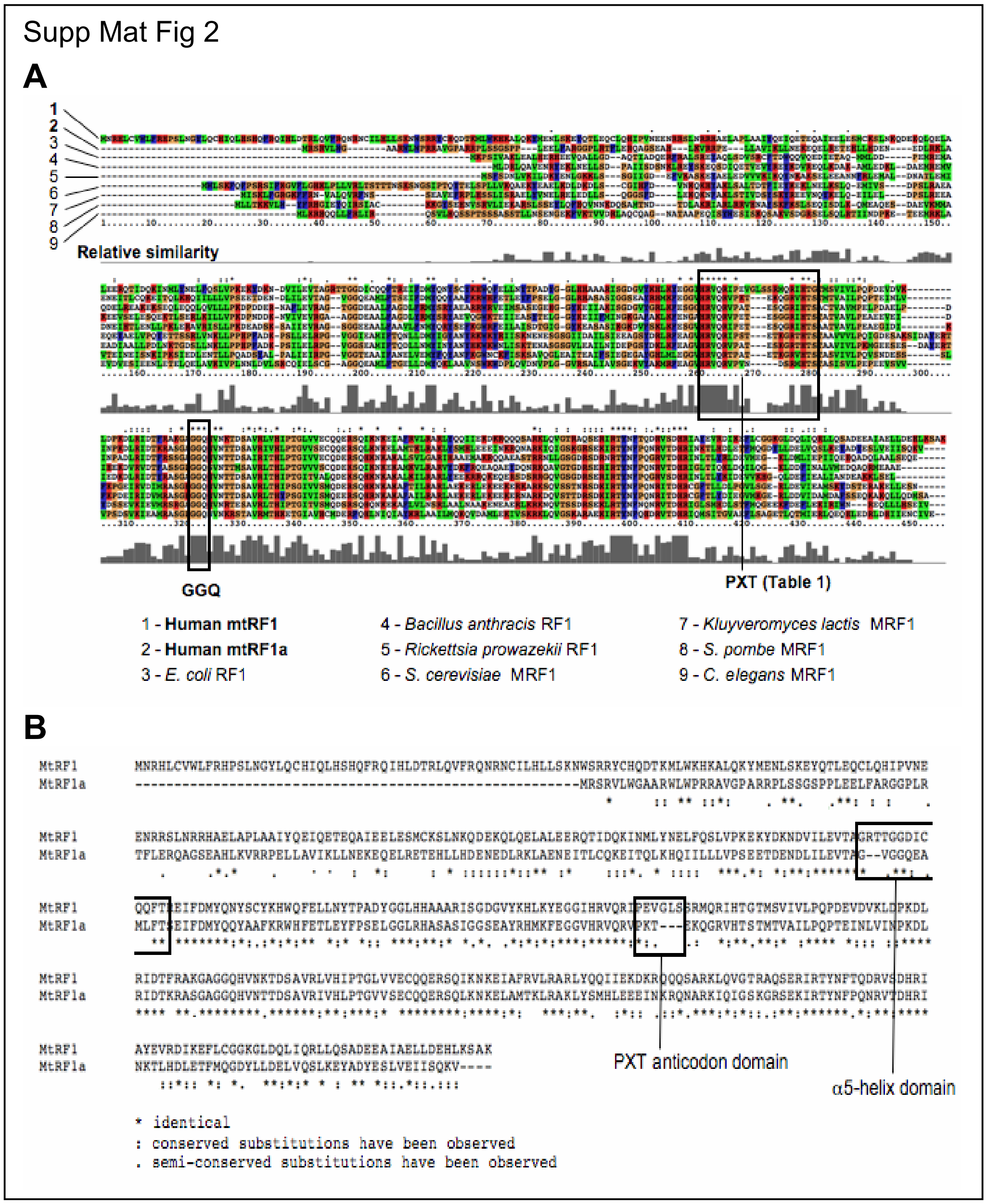

Figure S3. Optimisation of the translation release assay. The basic assay for translation release is described in Experimental Procedures. Optimisation was performed by varying the time for f[^{3}H]Met release from the ribosome (panel A) over 1 h. No increase in activity was observed beyond 40 min so this was selected as the optimal incubation time. Amounts of synthetic triplet (panel B) and purified human mtRF1a (panel C) were also varied, from 0-1 nmol and 0-100 pmol respectively. Synthetic stop codons were subsequently used at 400 pmol with 50 pmol of protein as amounts above these gave no great increase in activity. The effect on termination activity of increasing the synthetic stop codon from a triplet to 5 nucleotides was also examined (panel C) and was not found to improve efficiency of termination. Each panel includes SEM bars from over six independent experiments.

Supp Mat Fig 3

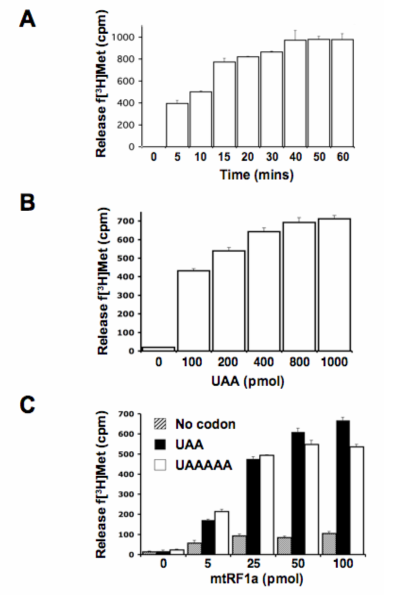

Figure S4. Expression of human mtRF1a in yeast mitochondria.

A. Westerns of mitochondria and post-mitochondrial supernatant from *S. cerevisiae* and *S. pombe* control strains and those producing human mtRF1a protein.

Both sets of data show that the human release factor is expressed, mitochondrially located and stable in yeast organelles, albeit at different levels. The left panels present mitochondria and post-mitochondrial supernatants from the *S. cerevisiae* Δ*mrf1Sc* NB345 strain transformed with plasmids producing either the mtRF1a or Mrf1Sc proteins. Nuclear encoded controls for efficient subfractionation include proteins located in the

mitochondrion (porin) or cytosol (phosphoglycerate kinase, PGK). These were only detected in the expected compartments. The right hand panel corresponds to a western of *S. pombe* mitochondria and post-mitochondrial supernatant from the wild-type NB205-6A with empty vector, or the Δ*mrf1Sp* strain NB329 with either empty vector, or one producing either HmtRF1a or Mrf1Sp. MtRF1a is detected mostly in the mitochondria of strain expressing the human cDNA, with a small fraction being present in the post-mitochondrial supernatant. The control antibody raised against the *S. cerevisiae* Arg8 protein (Steele *et al*., 1996) recognizes the *S. pombe* homologous protein Arg1Sp in mitochondria and probably a cross-reacting band in the post-mitochondrial supernatant.

B. Human mtRF1a can function in fission yeast mitochondria even at 36°C.

The Δ*mrf1Sp* strain NB329 or the isogenic wild type were transformed with either control vector or one encoding mtRF1a. Transformants patched on uracil-free medium were replicated onto galactose or glycerol/ethanol medium and incubated at 36˚C. Spectral analysis from Δ*mrf1* cells (grown on glucose at 36°C), wt and Δ*mrf1* cells producing HmtRF1a (grown on galactose at 36°C) confirmed restoration of cytochromes *b, c1* and *aa3* (right hand panel).

Supp Mat Fig 4

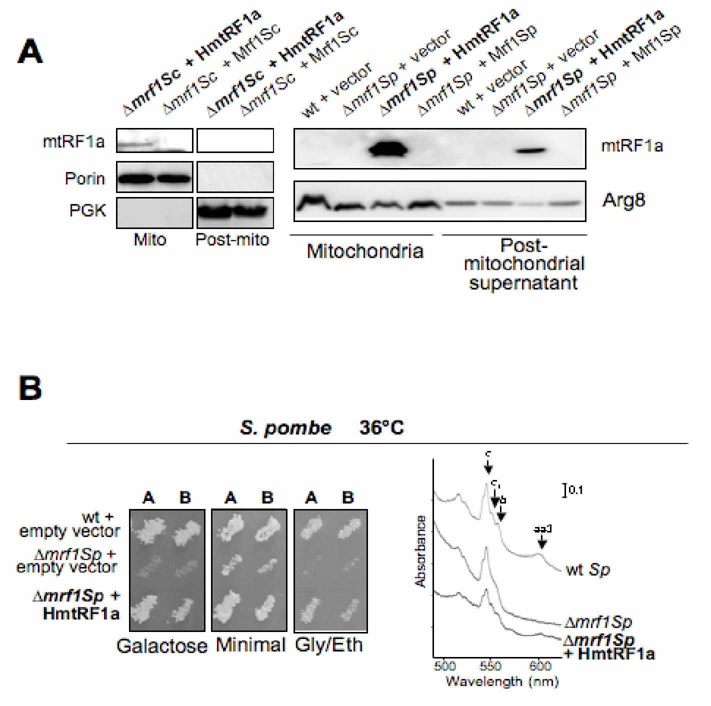

Figure S5. Human mtRF1 failed to compete for the ribosomal A site.

Release activity assays were programmed independently with mtRF1a or *E.coli* RF1. The addition of human mtRF1 did not affect the substrate release profile when the ribosomal A site was occupied by UAG or UAA. SEM bars were calculated from over six independent experiments.

Supp Mat Fig 5

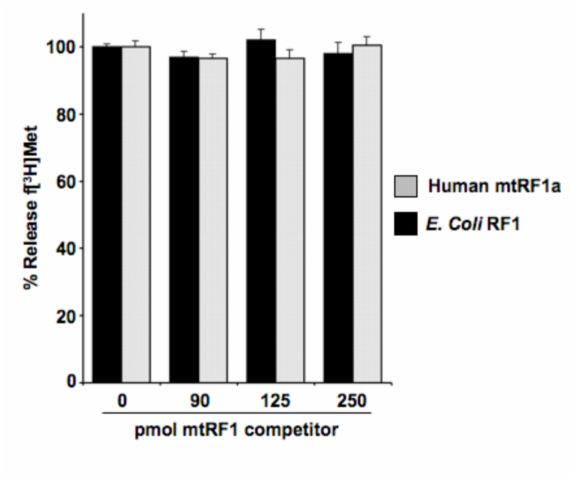

Supplement: Document S1. Supplemental Experimental Procedures, Supplemental References, One Table, and Five Figures [file mmc1.pdf]
